# Simple traumatic elbow dislocations; benefit from early functional rehabilitation: A systematic review with meta-analysis including PRISMA criteria

**DOI:** 10.1097/MD.0000000000027168

**Published:** 2021-11-05

**Authors:** Ilona Schubert, Peter C. Strohm, Dirk Maier, Jörn Zwingmann

**Affiliations:** a Clinic for Orthopedics and Trauma Surgery, Klinikum Bamberg, Germany; b Department of Orthopedic and Trauma Surgery, University of Freiburg Medical Center, Germany; c Clinic of Orthopedic and Trauma Surgery, Oberschwabenklinik Ravensburg, Germany.

**Keywords:** early functional rehablititation, simple elbow luxation

## Abstract

**Introduction:**

Elbow dislocation is the second most frequent joint dislocation after shoulder dislocation. They have a high relevance because they can result in subsequent damage and limitations in range of motion. The treatment options are controversially discussed.

The purpose of this systematic review and meta-analysis was to review the literature and analyze the evidence of early functional rehabilitation.

**Methods:**

A systematic literature search was performed via Ovid Medline, whereby 1645 publications were identified and evaluated in a stepwise approach. Of these publications 29 met the inclusion criteria of the authors and described simple elbow dislocations in 5765 patients.

Data from the studies and subgroups included were initially categorized descriptively in conservative and surgical primary therapies, in immobilizing (immobilization lasting 2 weeks or longer) and free-functional follow-up treatments, and those data were then extracted from each subgroup in absolutes. We then pooled these numbers into descriptive statistics to ensure their comparability. We determined the success rates from the numbers of excellent and good results of the specific used outcome scores.

**Results:**

The effect estimate of the conservative therapy's success rate was 84% and for surgical treatment 80% (*P* < .0001). The difference between the immobilizing treatment (78% success rate) and early-function therapy (83% success rate) was significant (*P* = .002).

In a subgroup analysis the success rate of conservative and immobilizing therapy was 79%, of conservative and early-functional therapy 91%, of surgical and immobilizing groups’ was 77% and of the surgical and early-functional therapies was 93%. The difference among the 4 treatment options was significant (*P* < .0001), as were differences between the 2 conservative groups (*P* < .0001) and between the 2 surgical groups (*P* = .044).

**Discussion:**

Conservative therapy is the dominant therapy. Regardless of the primary therapy chosen in simple elbow dislocations: early functional follow-up care seems to be superior to immobilizing therapy with a duration more than 2 weeks.

## 1 Introduction

Elbow dislocation is the second most frequent joint dislocation after shoulder dislocation in humans. Its incidence is about 6 per 100,000.^[1,2]^ Reports on concomitant fractures vary from 30% to 50%.^[3,4]^ The affected are usually adolescents and young adults^[1,5]^ and such injuries are most common in teenagers, but the median age is approximately 30 years.^[4,6,7]^

Elbow dislocations are caused by different accidents and are of varying frequency according to the individual's age. Sport-related injuries are most common among adolescents and young adults.^[1,4,6,7]^ High-energy traumas such as those associated with vehicular accidents are also common. Generally speaking, it is falling on an outstretched arm that triggers the dislocation.^[1,5]^ In their video analysis, Schreiber et al^[8]^ showed that acute elbow dislocations occur in relative extension regardless of the lower arm's position. Most common are falls onto the arm with an outstretched elbow, lower arm pronation, and abduction and ante version in the shoulder. Thus valgus, sprain, and supination forces exert their effects.

Older individuals suffer such injuries from traumas requiring less energy. One study found that more than a quarter of elbow dislocations occurred through falls on the ground level in individuals aged an average 55 years. Somewhat under three-quarters of the elbow dislocations were attributed to falls from a greater height or to a ground-level fall involving stronger forces; here the average age was 22.^[5,6]^

We differentiate simple from complex dislocations; associated fractures qualify a dislocation as being complex. Despite their classification, simple dislocations should not be underrated, as they may be accompanied by complex soft-tissue damage.^[1,2,9]^ Effective therapy depends on the type of injury. Complex elbow dislocations must be handled surgically and stabilized; the operative approach again depends on the precise nature of the injury.^[1,10,11]^ Simple dislocations, however, are mostly treated conservatively. Standard therapy involves temporary immobilization of the joint following closed repositioning and stepwise re-mobilization after thorough reassessment of the joint's stability. Surgical correction is indicated in patients with a tendency to re-luxate or where the joint is instable, as well as in case of a purely ligament dislocation to prevent persisting instability and its sequel.^[1,10,12,13]^

Complex elbow dislocations tend to entail a worse outcome than simple dislocations.^[1]^ Nevertheless, the latter can lead to secondary damage such as having lasting difficulty making certain movements, or chronic instability.^[3,4]^

The main aim of the present project was to collate and discuss the published material on elbow dislocations by carrying out a thorough and systematic literature search. Those results were then subjected to a meta-analysis of individual investigations according to their epidemiological specifications and the therapy introduced, and their outcomes then compared. In so doing, we hope to determine the evidence of evidence-based treatment standards for simple elbow dislocations. Surgical and conservative primary therapies requiring immobilization, versus the early-functional follow-up care will be compared.

## 2 Methods

This systematic review and meta-analysis were based on an OVID-based literature search. Here, all published clinical studies on simple elbow dislocations were found in the following databases: MEDLINE, MEDLINE preprints, EMBASE, CINAHL, Life Science Citations, the British National Library of Health, and the Cochrane Central Register of Controlled Trials (CENTRAL). MEDLINE served as our data base, which included publications dating from 1947 through 1.1.2017.

Our search queries followed the strategy illustrated in Table 1. We first filtered those publications addressing the elbow or elbow joint, joint instability and dislocation. Our final literature search result included 1645 articles. An ethic committee votum was not needed for this study because no patient was involved and it was only a meta-analysis of the literature.

**Table 1:** Ovid search strategy.

Search strategy	
	Entry
1	Elbow joint/
2	Elbow/
3	Joint instability/
4	Dislocations/
5	1 or 2
6	3 or 4
7	5 and 6
8	((elbow^∗^ or ulnohumeral or radiohumeral) adj3 (dislocat^∗^ or sublux^∗^ or instab^∗^ oder unstab^∗^)).tw.
9	7 or 8
10	Treatment outcome/
11	9 and 10
12	Treat^∗^.tw.
13	10 or 12
14	9 and 13
15	exp Animals/ not Humans/
16	14 not 15

The abstracts of the 1645 publications were studied by 2 independent reviewers. Studies with their abstracts not meeting the exclusion criteria were manually analyzed, compared, and included or excluded from our analysis according to the following criteria.

### 2.1 Inclusion criteria

We included all the prospective and retrospective clinical follow-up studies and those with subgroups categorized in terms of the therapy of simple elbow dislocation and its outcome.

### 2.2 Exclusion criteria

- General overview papers and review articles (132)
- Investigations on dislocations involving any kind of fracture (869)
- Follow-up periods lasting <12 months (67)
- Follow-up rates <70%
- Case reports (89)
- Biomechanical, radiological, or surgical-technical studies (397)
- Any articles not in German or English (62)

The first exclusion criterion to come to light was always the only 1 that was taken into account.

**Figure 1:**
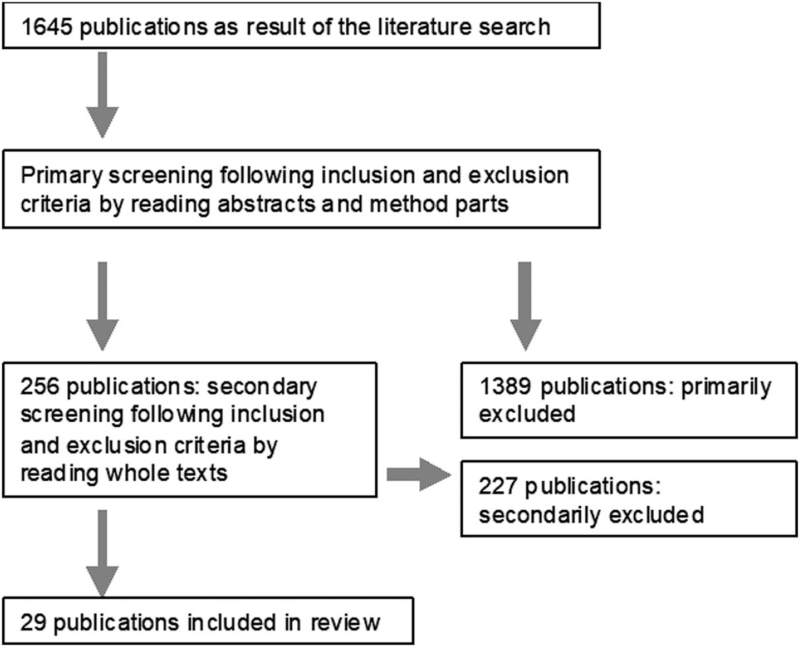
Selection process. The flowchart showing selection process for the publications included according to PRISMA.^[14,15]^

The various phases of our systematic overview followed Preferred Reporting Items for Systematic reviews and Meta-Analyzes in a flow diagram as indicated in Figure 1.^[14,15]^

The 29 publications included are shown. One Study had a level of evidence of II, 6 studies a level of evidence of III und 22 studies had a level of evidence of IV.

### 2.3 Subgroup formation

The 29 publications we included were then classified in subgroups of patients diagnosed with simple dislocations according to their age range and specific treatment, yielding 36 subgroups. The studies we divided were numbers 1, 2, 4, 5, 8, 16, 24; (see Table 2).

**Table 2:** The 29 publications included.

Nr.	Author	Year	Journal	Reference
1	Fowles et al	1984	Am J Bone Joint Surg.	^[16]^
2	Borris et al	1987	Acta Orthop Scand.	^[17]^
3	van der Ley et al	1987	Neth J Surg.	^[18]^
4	Josefsson et al	1987	Am. J Bone Joint Surg.	^[19]^
5	Josefsson et al	1987	Clin Orthop Relat Res.	^[20]^
6	Mehlhoff et al	1988	Am. J Bone Joint Surg	^[21]^
7	Maggi et al	1992	Chir Organi Mov	^[22]^
8	Riel et al.	1993	Unfallchirurg	^[23]^
9	Schippinger et al	1999	Langenbecks Arch Surg.	^[24]^
10	Ross et al	1999	Am J Sports Med.	^[25]^
11	Jupiter et al	2002	Am J Bone Joint Surg.	^[26]^
12	Olsen et al	2003	J Bone Joint Surg Br.	^[27]^
13	Eygendaal	2004	Am J Sports Med	^[28]^
14	Devnani	2004	Singapore Med J	^[29]^
15	Mahaisavariya et al	2005	Clin Orthop Relat Res	^[30]^
16	Maripuri et al	2007	Injury	^[31]^
17	Duckworth et al	2008	J Shoulder Elbow Surg.	^[32]^
18	Jeon et al	2008	Keio J Medicine	^[33]^
19	Micic et al	2009	Intern Orthop.	^[34]^
20	Kesmezacar et al	2010	Acta OrthopTraumatol Turc.	^[35]^
21	Anakwe et al	2011	Am J Bone Joint Surg.	^[36]^
22	Jockel et al	2013	Am J Hand Surg.	^[37]^
23	Adas et al	2014	Intern Orthop.	^[38]^
24	Iordens et al	2015	Br. J Sports Med.	^[39]^
25	Schnetzke et al	2015	J Orthop Surg. Res.	^[40]^
26	Schreiber et al	2015	Am. J Hand Surg	^[41]^
27	Mayne et al	2015	J Shoulder Elbow Surg.	^[42]^
28	Modi et al	2015	Injury J.	^[43]^
29	Sofu et al	2016	J Intern. Orthop	^[44]^

The 29 publications included are shown in Table 2. 1 Study had a level of evidence of II, 6 studies a level of evidence of III und 22 studies had a level of evidence of IV.

The data below were extracted from the studies we included:

1. Name or names of the authors, journal name, year of publication, type of study
2. Patient numbers or number of elbow dislocations investigated
3. Epidemiological data (age, gender distribution)
4. Follow-up as an average in months
5. Dislocation direction and instability
6. Type of therapy and follow-up treatment
7. Therapy outcome
  a. Elbow-specific Outcome Scores
  b. Range of movement measured in angular degree (flexion/extension, range of movement, pronation/supination)
8. Complications
  a. vessel-nerve lesion/s (yes/no)
  b. indications of arthrosis (yes/no)
  c. heterotopic ossification (yes/no)
  d. redislocation (yes/no)
  e. elbow pain (yes/no)
  f. weakness (yes/no)
9. Follow-up surgery: type and time span until the surgery

We extracted the elbow-specific outcome scores categorically: Both the average values of the respective score, and the number of patients with excellent, good, moderate, and poor results were noted, if indicated. We included the scores below:

- Mayo Elbow Performance Score
- Broberg and Morrey Index
- Morrey Score
- Functional criteria according to Roberts (cR)
- The Disabilities of the Arm, Shoulder and Hand Questionnaire (DASH, Quick-DASH)
- The Oxford-Elbow Score (OES)
- American shoulder and Elbow surgeons’ outcome (ASES)
- Modified Andrew's Elbows Scoring System (MEASS)
- Elbow Function Assessment Scale (EFAS)

### 2.4 Quality assessment

We determined the level of evidence of all the publications included in this report^[45]^ as well as the Coleman Methodology Score (CMS). The CMS is a score consisting of 10 criteria to assess the methodology of individual studies: study size, mean follow-up, number of surgical procedures, type of study, diagnostic accuracy, description of surgical procedure, postoperative rehabilitation, outcome measures, outcome assessment, and selection process.^[46]^ The points that can be achieved range from 0 to 100 points, with 100 points being the best possible result, that is, a study design that largely avoids the influence of chance, different biases, and confounding factors.^[46]^ The Coleman Methodology Score is an established mean to evaluate the studies’ quality.^[47,48,49]^

### 2.5 Data processing

Data from the studies and subgroups included were initially categorized descriptively in conservative and surgical primary therapies, in immobilizing (immobilization lasting 2 weeks or longer) and free-functional follow-up treatments, and those data were then extracted from each subgroup in absolutes. We then pooled these numbers into descriptive statistics to ensure their comparability.

The tables and descriptive ratios were created by relying on Microsoft Excel 2003.

In step 2 of this assessment, we conducted a meta-analysis of the studies and subgroups: we compared the success rates of individual subgroup populations according to their treatment regime. We determined the success rates of most of the studies (namely, 20 subgroups) from the numbers of excellent and good results of the aforementioned specific outcome scores as the quotient. For those studies not assessing therapy results by relying on elbow-specific outcome scores (14 subgroups), but instead only reported basic data such as range-of-motion (given in), pain (as yes/no) and instability (given as the direction), we calculated a mean success rate to feed into our meta-analysis. By relying on the Mayo Elbow Performance Score, which incorporates the rage of motion, amount of pain instability and everyday function, the success rate was calculated from the quotient of the movement arcs and the norms and the proportion of study participants not suffering from pain.

Data assessment and a forest plot were facilitated by the “Comprehensive meta-analysis”- Version 2 program.

This analysis took possible study effects into account, and a random-effects model was used for statistical analysis. The protocol was not preregistered on an open website. For all meta-analyses, tests for differences between subgroups were performed. The results are given as *P* values. Significant *P* values indicate an existing difference in the summary intervention effects between subgroups. Furthermore, taking the quantity of statistical heterogeneity between studies into account, values of *I*^2^ were calculated for each subgroup.

## 3 Results

### 3.1 Descriptive assessment

The 29 studies we included describe simple elbow dislocations in 5846 patients. Of those, 81 children (<16 years; range 5–16 years) had to be excluded, leaving us with 5765 patients (≥16 years, range 7–88 years) to analyze; in the latter, there were 6 studies with groups made up of patients varying in age (^[18,22,27,29,30,36]^ - see Table 2), meaning that the adult group also contains very few patients under age 16. Nevertheless the weighted age average (product of the studies mean age multiplied by the percentual proportion of patient numbers age) was 398 years with a gender ratio of 093 men/women. Average follow-up period was 96 months.

#### 3.1.1 Conservative treatment

n = 5636, mean age 40 years, gender ratio 0.91 men versus women, mean follow-up 103 months. 86% posterior and posterolateral dislocation, 4.6% not specified, 3.2% posteromedial dislocation.

##### 3.1.1.1 Conservative treatment via immobilization

n = 516, mean age 36.9 years, gender ratio 1.05, mean follow-up 62 months, 86% posterior and posterolateral dislocation, 6.7%, dislocation not specified and 4% posteromedial dislocation. Mean period of immobility of 2.7 weeks. Five percentage secondary surgeries.

##### 3.1.1.2 Conservative treatment via early functional therapy

n = 242, mean age 38 years, gender ratio 1.55, mean follow-up 28 months. 93% posterior and posterolateral dislocation, 2.6% lateral dislocation, 2.6% not specified. Average period of immobility was very brief (0.35 weeks, or about 3 days). The splinting period was described as the time when elbow movements were being practiced although still outfitted with a splint, which took an average of 9.4 days. Eight percentage secondary surgeries.

#### 3.1.2 Operative treatment

n = 129, mean age 37 years, gender ratio 2.3, mean follow-up 32 months. Fifty one percentage posterior and posterolateral dislocation, 33% not specified, 26% had dislocation >3 weeks.

Mean time to surgery 7.2 days. Seventy five percentage primary ligament repair, 16% secondary ligament repair.

##### 3.1.2.1 Operative treatment via immobilization

n = 91, mean age 37 years, gender ratio 1.5, mean follow-up 35 months, 73% posterior and posterolateral dislocation, 11% not specified, 3.5% medial dislocation, 31% had dislocation >3 weeks.

Mean time to surgery 49 days. Mean period of immobilization 3.2 weeks. Sixty seven percentage primary ligament repair (12% lateral ligament complex, 8% lateral and medial ligament complex, 47% not specified), 23% secondary ligament repair. Four percentage secondary surgeries.

##### 3.1.2.2 Operative treatment via early functional therapy

n = 38, mean age 37 years, gender ratio 4.4, mean follow-up 29 months, 7% posterior and posterolateral dislocation, 87.5% not specified, 13% had a dislocation >3 weeks.

Mean time to surgery 13 days. Mean time of immobilization 0.55 weeks, mean splinting period 34 days. Ninety five percentage primary ligament repair (39% lateral ligament complex, 14% medial ligament complex, 47% lateral and medial ligament complex). Eight percentage secondary surgeries.

### 3.2 Outcome assessment

Due to limited outcome data in the studies Nr. 27^[42]^ and 28^[43]^ (see Table 2), those were only used for descriptive epidemiological assessment.

Considering the outcome scores, we identified a total of 421 excellent or good, and 72 fair or poor treatment results. The success rate was 83% taking all studies and 887 patients into account.

The specific outcome scores after conservative treatment yielded 373 excellent or good and 68 fair or poor results. The success rate among adults treated conservatively was 84%. The conservatively-treated, immobilized group revealed 238 excellent or good, and 59 fair or poor results; their success rate was 79%. The conservatively-treated, early-functional group had 135 excellent or good and 9 fair or poor results. Their success rate was 91%.

The surgically-treated group of adults had 48 excellent or good and 4 fair or poor results; their success rate was 80%. The subgroup immobilized postoperatively had 13 excellent or good and 2 fair or poor results and a success rate of 77%. The postoperative, early functional subgroup had 35 excellent or good and 2 fair or poor results with a 93% success rate.

Considering the range of motion, a total of 215 patients reported movement restrictions after treatment, although not every study provided mean values to describe such limitations.

The group treated conservatively contained 136 individuals (18%) with restricted movement. The mean amounts of movement were: extension-flexion arc (E-F arc) 130°, extension deficit (ED) 9°, flexion 139°, pronation 83°, supination 83°, pronation-supinations-arc (pro-sup arc) 166°. Seventy six patients (10% of those treated conservatively) presented an ED <10°, 42 (6%) had an ED measuring 10 to 30°, and 9 patients (1%) suffered an ED ≧30°. Twenty patients (3%) had a flexion deficit (FD) under 10°, and 3 (04%) had 1 between 10 and 25°.

In the conservatively-immobilized subgroup, 115 patients (22%) suffered from restricted movement. Their degrees of movement were: E-F arc 131°, ED 11°, flexion 139°, pronation and supination 84°, respectively, pro-sup arc 169°. Sixty four patients (12% of the subgroup) presented less than a 10° ED, whereas the ED measured between 10 and 30° in 29 (6%), and ≧30° in 9 (2%) patients. Eleven patients (2%) had an FD <10°; 9 patients (2%) an FD between 10 und 25°.

There were 21 patients in the conservatively-treated, early functional subgroup with movement restrictions, or 9% of that subgroup. Their mean E-F arc was 136°, the ED 5°, flexion 140°, pronation 86°, supination 87°, the pro-sup arc 172°. Twelve patients (5%) had an ED <10° and a deficit between 10 and 30° (5%), no patient has a deficit ≧30°. Nine patients presented an FD <10° (4%).

In the group treated surgically, 20 patients or 16% reported movement restrictions. Their mean amounts of movement were: E-F arc 113°, ED 19°, flexion 130°, pronation 79°, supination 81°, pro-sup arc 159°. Eleven patients (9%) had an ED <10°, 32 (25%) an ED between 10 and 30°, and 23 (18%) an ED ≧30°. One patient (0.8%) suffered an FD <10°.

Regarding the operatively-treated, postoperatively immobilized subgroup: 20 patients (22%) reported movement restrictions. Their mean amounts of movement were: E-F arc 111°, ED 21°, flexion 130°, pronation 79°, supination 80°, pro-sup arc 159°. Seven patients (8%) had an EF <10°, 17 patients (19°) an ED between 10 und 30°, and 22 (24%) suffered an ED ≧30°. One patient (1%) had an FD <10°.

In the operatively-treated, postoperatively early functional subgroup the mean amounts of movement were: E-F arc 117°, ED 14°, flexion 131°, pronation 78°, supination 83°, pro-sup arc 160°. Four patients (11%) had an ED <10°, 15 (39°) an ED between 10 und 30°, and 1 (3%) an ED ≧30°. There was no data available on the absolute numbers of patients with movement restrictions.

### 3.3 Descriptive assessment of complications

A total of 186 patients reported persistent pain, or 20% of all the adults. Only the absolute numbers of patients with pain were reported, but not the mean amounts of pain according to various pain scales. Heterotopic ossification was noted in 304 adults (33%). Neurovascular deficits after dislocation were diagnosed in 58 patients (6%), redislocations in 3 (03%), and degeneration or signs of arthrosis in 67 patients (7%).

Pain was attributed to 167 patients (22%) in the conservative group; 241 (32%) presented heterotopic ossification, 44 (6%) suffered neurovascular deficits. Two patients (0.3%) had a redislocated elbow, and 53 (7%) degeneration or signs of arthrosis.

The immobilized subgroup contained 159 patients (30%) reporting pain, 193 (37%) developed heterotopic ossification, 26 (5%) had a neurovascular deficit, 1 (0.2%) suffered a redislocation, and 28 (5%) presented degeneration or signs of arthrosis. Eighteen (3%) patients reported weakness in the affected arm.

The early functional subgroup contained 8 patients (3%) reporting pain, 48 (20%) heterotopic ossification, 10 (4%) a neurovascular deficit, and 1 (0.4%) suffered a redislocation. Degeneration or signs of arthrosis were diagnosed in 25 patients (10%).

In the surgical group there were 19 patients (15%) reporting pain, 63 (49%) developed heterotopic ossification, 14 (11%) a neurovascular deficit, 1 patient (0.8%) suffered a redislocation and 14 patients (11%) developed degeneration or signs of arthrosis.

Regarding the operatively-treated, postoperatively immobilized subgroup: 16 (18%) patients reported elbow pain, 42 elbows (46%) revealed heterotopic ossification, and 5 patients (5%) complained of a neurovascular deficit. One patient (1%) suffered a redislocation, and 4 (4%) presented degeneration or signs of arthrosis.

In the operatively-treated, postoperatively early functional subgroup there were 3 patients (8%) reporting pain, 21 (55%) presenting heterotopic ossification, nine (24%) developed a neurovascular deficit, and 10 patients (26%) were diagnosed with degeneration or signs of arthrosis. No patient suffered a redislocation.

## 4 Results of the meta-analysis

### 4.1 Simple elbow dislocation in adults: comparing outcomes of conservative to surgical therapy

The effect estimate of the conservative therapy's success rate was 0.84 (84% success rate); that of surgical treatment 0.80 (80% success rate).

The conservative group's statistic heterogeneity revealed an *I*^2^ value of 69.8%. The surgical group's *I*^2^ value of 0% exhibited heterogeneity. The “conservative” subgroup's statistic heterogeneity was significant, while the surgical subgroup's was not.

**Figure 2:**
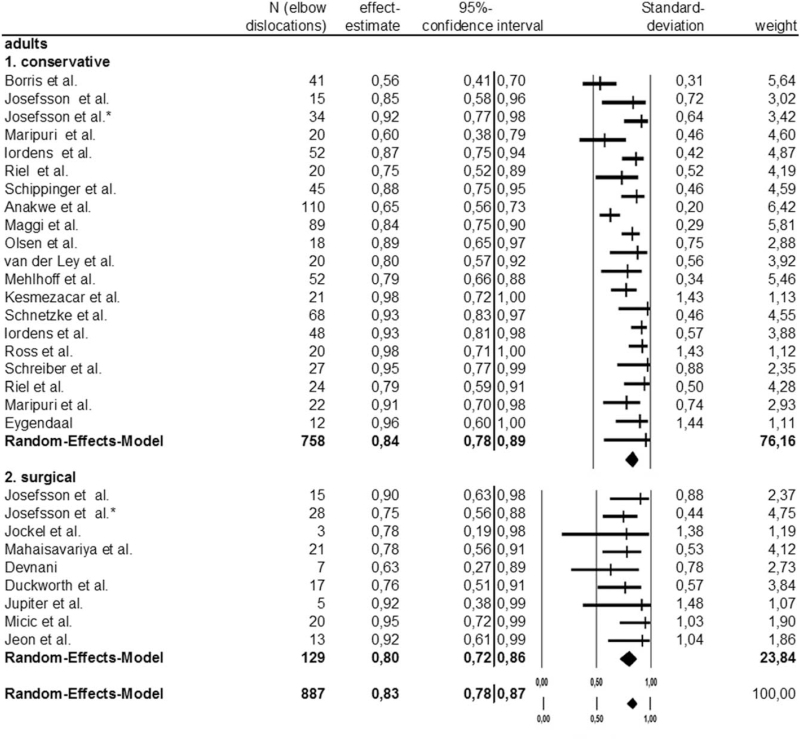
Forestplot and Meta-analysis of simple elbow dislocation in adults: comparison of conservative to surgical therapy.

The difference between the conservative and surgical therapy groups was significant (*P* < .0001) Figure 2.

### 4.2 Simple elbow dislocation in adults: comparison of outcomes of immobilizing treatment to early-function therapy

The immobilizing subgroup's statistic heterogeneity was with an *I*^2^ value of 54.5% potentially moderate, while the early-functional subgroup's measuring 0% was thus inessential. The immobilizing subgroup's statistic heterogeneity was significant; the early-functional subgroup's was not. The difference between the immobilizing treatment and early-function therapy subgroups’ simple elbow dislocations was significant (*P* = .002).

**Figure 3:**
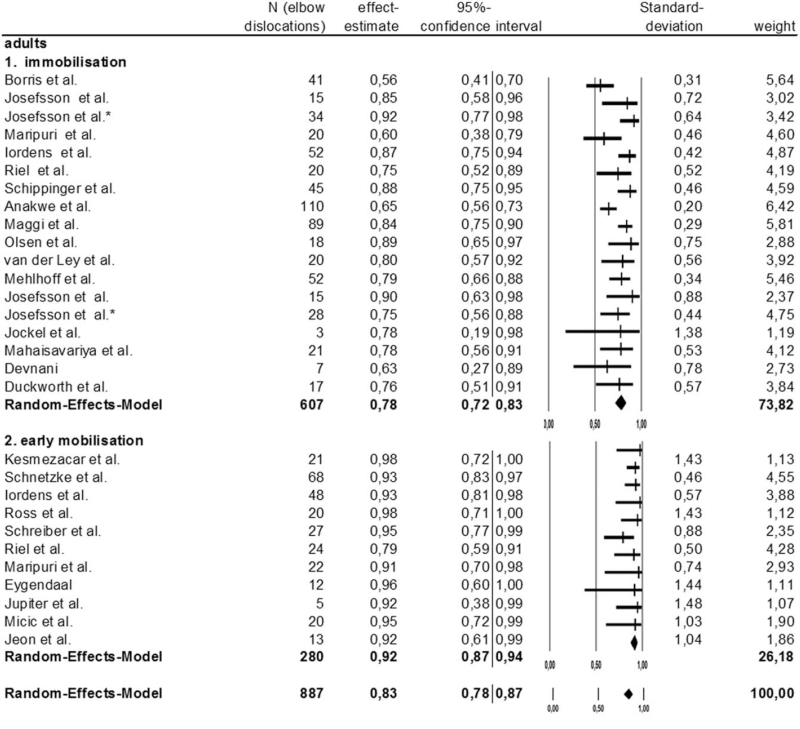
Forestplot and Meta-analysis of simple elbow dislocation in adults: comparison of immobilizing treatment to early-functional therapy.

The immobilizing subgroup's effect estimate measured 0.78 (0.78% success rate), the early functional group's 0.83 (83% success rate) Figure 3.

### 4.3 Simple elbow dislocation: comparison across subgroups of therapeutic outcomes

The effect estimate of conservative, immobilizing therapy was 0.79 (79% success rate), of conservative, early-functional therapy 0.91 (91% success rate), the surgical and immobilizing groups’ was 0.77 (77% success rate); the surgical and early-functional therapies was 0.93 (93% success rate).

The conservative, immobilizing therapy‘s statistic heterogeneity revealed an *I*^2^ value of 68.61% (essential). The 3 other treatment options *I*^2^ value (ranging from 0%–1.11%) was inessential. Only the conservative, immobilizing therapy revealed significant statistic heterogeneity.

**Figure 4:**
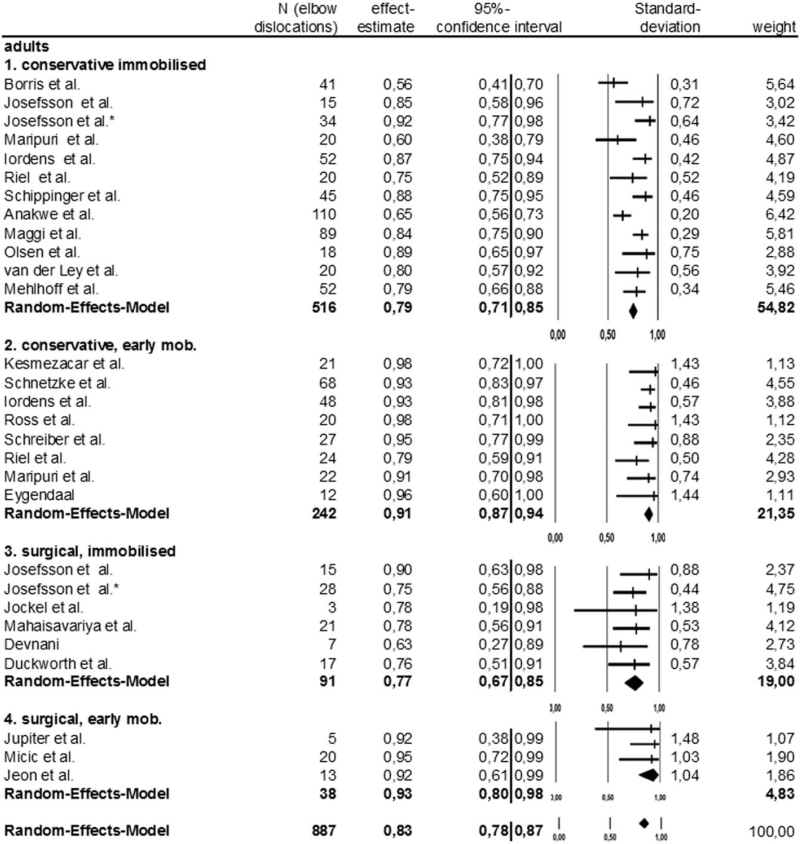
Forest plot and Meta-analysis of simple elbow dislocation in adults: therapy comparison across subgroups.

The difference among the 4 treatment options was significant (*P* < .0001), as were differences between the 2 conservative groups (*P* < .0001) and between the 2 surgical groups (*P* = .044). Not significant was the difference between the conservative and surgical groups after immobilizing follow-up therapy (*P* = .668), nor was the difference between conservative and surgical groups that had undergone early-functional follow-up therapy (*P* = .686) Figure 4.

### 4.4 Quality assessment of the included studies

To assess the quality of the studies we included, we employed the Coleman Methodology Score (CMS) and Level-of-Evidence Rating System. Four investigations were prospective, while the remaining 25 were of retrospective design. One was a prospective cohort study; 6 were case-control studies. Most of the 22 studies were case series with evidence level IV - a moderate evidence level. The investigations’ quality was further quantified via the CMS. The 29 studies attained an average CMS score of 53.97+/−11 (48–90) of a maximum 100 points, which no study achieved. The conservative, immobilized subgroup earned the highest CMS at 63.2+/−10.0 points, while the surgical, early-functional follow-up care subgroup scored the lowest at 49.7+/−1.7 points. As most of the studies included were case series, the CMS part B3 is most relevant, as it provides evidence of potential distortion, that is, in selecting patients. Here the average was 11.9 +/−3.1 earned of a maximum 15 points; thus the likelihood of distortion is low.

## 5 Discussion

Aim of this study was to provide as thorough an overview as possible of the latest data on the treatment and epidemiology of simple elbow dislocations and analyze the evidence of early functional rehabilitation.

To be able to conclude therapy recommendations for simple elbow dislocation, we divided the included studies into therapy subgroups according to these categories: “conservative and immobilized,” “conservative and early-functional,” “surgical with early-functional follow-up care” and “surgical with immobilized follow-up care”. The analysis of the outcome scores resulted in success rates for each treatment option. Despite the heterogeneity, the outcome scores we fed into our meta-analysis reflect comparability thanks to them sharing the same scoring system. The comparability of total results from Mayo Elbow Performance Score and Broberg and Morrey Index were verified by Turchin et al already 20 years ago.^[50]^ We applied the Random Effects Model for our meta-analysis, as it takes statistic heterogeneity into account. All in all, the success rate was good with 83% for all included dislocations.

Nevertheless, although being called simple, these dislocations should not be underestimated, as they can be accompanied by complex soft-tissue injuries.^[1,2,9]^ For a positive outcome, prompt corrective therapy is essential to minimize sequel such as persistent pain, chronic instability, or permanently restricted movement.^[3,4]^

Many working groups have addressed the obvious question as to what constitutes the optimum therapy for simple elbow dislocation. The multicentric, randomized, controlled study by Lordens et al^[39]^ showed that conservative early-functional treatment was superior to conservative immobilizing therapy (3 weeks of immobility) over the short term. The early-functionally-treated patients revealed a better outcome in terms of their range of motion, working capacity, and clinical scores without carrying a higher risk of redislocation or instability. However, after a year's follow-up there was no significant difference between the 2 groups. In their 2012 review, Taylor et al^[7]^ compared the studies of Rafai et al from 1999^[53]^ to that of Josefsson et al from 1987^[19]^ and reported that the early-functional group's condition was better in mobility, after a year's follow-up than the immobilized group's but the difference was statistically insignificant. In their 2009 review, De et al^[3]^ described improved range of motion, less pain, better functioning, and briefer treatment durations in their early-functional group than in their immobilized cohort. Maripuri et al.^[31]^ also demonstrated better functional outcomes and an earlier return to work in their early-functional group without raising the risk of instability or redislocation. We also detected no increase in the tendency for redislocation in the early-functional group. At any rate, the redislocation rate was negligible in all 4 groups (≤1% in each).

With the conservative-immobilizing being the largest subgroup, and the conservative-early-functioning subgroup as second largest 1, the conservative therapy still is the most common primary treatment method. Consistent with the literature, we conclude from our findings a significant better outcome comparing conservative early-functional (success rate 91%) to conservative-immobilizing treatment (success rate 79%). Most of the studies in the conservative early-functional group are more recent than those in the conservatively-treated immobilized group, indicating that the therapy is trending to early-functional regimes. Nevertheless, considering our subgroups’ patient numbers- 516 immobilized versus 242 early-functional - the vast majority of patients treated conservatively also experienced an immobilizing follow-up therapy with a mean period of immobility of 2.7 weeks. In the conservative-immobilized group the mean extension deficit was 11°; just 2% of patients suffered an extension deficit of ≧30°, the mean bend capacity was 139°. For effective mobility for daily activities an E-F arc of at least 0–30°-100° is considered necessary.^[4,56,57]^ Thus we can assume that, despite the poorer success rate, this subgroup maintained very good everyday mobility.

Although 36% of the elbows labeled instable in the conservative early-functioning group, the high success rate may advocate for patients with minor instabilities, or a good surgery-free treatment option for minor instability. The latter is, however, compromised by the relatively high rate of secondary tendon interventions.

Beyond this, the early-functional group with primary surgical therapy also showed significantly better results than the postoperative immobilized group (success rates 93% vs 77%). Surgical therapy for simple elbow dislocation is not as nearly as highly regarded as it is for complex ones: according to Grazette et al,^[54]^ the percentage of patients who underwent surgery for simple elbow dislocation within 4 years was only 4%. In our study the surgical approach as well played a minor role, as revealed by the numbers in the studies we included: 129 surgically-treated patients versus 758 handled conservatively. However, surgical treatment must not be disregarded because closed reposition is the standard approach, in patients showing a redislocation tendency or suffering from instability even in conjunction with simple ligamentary dislocation, surgical therapy is indicated.^[1,10,11,12,13]^ The extent to which early surgical therapy is beneficial is under discussion. Joseffson et al^[19]^ reported no significant difference in movement outcomes in patients treated conservatively compared to those who underwent primary ligament reconstruction for simple, medially-unstable elbow dislocation, but did note a negative trend concerning the surgical approach. The review of Grazette et al from 2017^[54]^ reported no significant advantage from the early surgical ligament repair of damaged soft-tissue structures over early-functional treatment after stable repositioning. In patients presenting pronounced instability, surgical therapy is indicated, although the authors report no difference between early and later surgical therapies.

Mostly primary ligament repair takes place within the first 14 days, as done later it would be problematic due to rapidly-developing post-injury ligament fibrosis.^[12]^ Contrary to this statement Hackl et al^[2]^ report in their meta-analysis, that tendon reconstruction is equally effective from months to years after an acute ligament suture. We need to consider Joseffson et al^[19]^ critically nowadays, as surgical techniques have improved dramatically since 1987, and very recent studies have demonstrated surprisingly good results after surgical management.^[32,33,34]^ In our study 75% percent of the included elbows with primary surgical therapy underwent primary tendon reconstruction. The brief time to surgery with a little bit over a week can be considered positively. Secondary ligament repair was rather rarer. This may be due to the brief average period between dislocation and surgery, as secondary ligament surgery is usually carried out when a patient's surgery has been delayed, in which case the original tendon structures often become scarred.^[2,58]^

Twenty percentages of the conservatively-treated immobilized elbows were diagnosed with instability after dislocating, while 36% of those in the early-functional group were instable. 4.5% of the primarily conservative-immobilized patients secondarily underwent surgery (83% of those stabilizing surgery). This implies that only under a quarter of the instable dislocations had to secondarily be stabilized surgically. Although in the conservative early-functional subgroup 8% eventually had to undergo secondary surgery, nearly twice the percentage as in the conservatively-treated immobilized subgroup, there is evidence in the literature of better outcomes after early-functional therapy without any additional risk of instability or reluxation.^[31,39]^ In our study the risks of secondary surgery were similar in both immobilized and both early-functional groups. This seems to indicate that the necessity of secondary surgery depends not on the primary therapy (conservative versus surgical) but more likely on the choice of follow-up care (early-functional versus immobilized). The 100% rate of instability was predictable in the surgically-treated, early functional group. Their very good success rate (93%) supports this therapy option for the right indication. On the other hand, no instability was explicitly described in conjunction with 28% of the surgically, immobilized followed-up group. We cannot say how many of those operations were carried out for a correctly-determined indication. However, the 77% success rate puts this treatment option in doubt. Concluding, this means that not every elbow designated as instable, that has been successfully set, should be operated on, as the success rates revealed that especially the conservatively-treated, early functional group had an excellent treatment result (91%).

Whereas in the conservative groups the indication for secondary surgery, although rare overall, mostly based on instability, in the surgical groups’ secondary surgeries mostly included arthrolysis. To summarize: it is essential that the physician examines the patient closely promptly after an accident to rule out or diagnose any instability to enable surgery when indicated and thereby optimize the final outcome. Depending on the degree of instability and individual patient factors, a decision must be made for or against surgery and the surgical approach. MRT examinations are also worthwhile to determine the extent of soft-tissue damage or detect any cartilage injuries bone damage not visible on X-ray.^[12]^

Regardless of the primary kind of therapy, if correctly indicated, also in the surgical groups the early-functional follow-up showed significantly better results than the immobilizing treatment. Only 8% experienced pain, further supporting their excellent outcomes. However, 55% developed heterotopic ossification, making them the group with the highest proportion of tissue ossification. Our data did not enable us to say whether this observation is related to the severity of the injuries in the surgical groups (more cases of instability) than in the conservative groups, or whether it is associated with the operations themselves. Debatable is the extent to which their good outcome could be improved via medical prophylaxis for heterotopic ossification, that is, Indometacin, or whether the secondary-surgery rate (e.g., arthrolysis) might be reduced by having patients take an ossification prophylaxis.

## 6 Conclusion

According to the patient numbers in the subgroups categorized by treatment, conservative therapy is the dominant therapy in simple elbow dislocations and remains the treatment standard.^[1,59]^ After closed repositioning followed by further conservative therapy, we found that early-functional follow-up care entailing absolute immobilization for less than 2 weeks was significantly better than immobilized follow-up therapy.

Despite the excellent outcomes observed after conservative therapy, the indication for surgical stabilization should be kept in mind for patients with simple dislocation, especially in cases of severe instability. We observed that the outcomes of surgical therapy and early-functional follow-up care were nearly equal to those associated with conservative, early-functional treatment (there was no significant difference), and significantly better than those of surgical therapy and postoperative immobilization. What seems most important is determining the correct indication, as the success rate of the surgical, early-functional therapy supports the position that modern surgical procedures in conjunction with the right indication can result in very good outcomes - even in patients with more serious soft-tissue injuries. Nevertheless, patients must be well informed of the risks of arthrolysis, as the surgical groups required the most secondary interventions, and more heterotopic ossifications were diagnosed in the 2 surgically-treated groups than in those treated conservatively. The total proportion of secondary surgical interventions in all 4 treatment groups was under 10%.

With success rates of 91% in the conservative group and 93% in the surgical group, early-functional follow-up care yielded a very good functional outcome and in addition we detected no increase in the tendency for redislocation in the those groups. Nevertheless, brief immobilization after the injury or operation might provide better pain relief and wearing or theses during early-functional therapy, might enable certain movements and thus early exercise.

## Acknowledgment

The authors thank Mrs. Buroh of the Library of the Medical Center - University of Freiburg very much for the support in performing the search strategy and the literature search.

## Author contributions

**Conceptualization:** Peter C. Strohm, Jörn Zwingmann.

**Data curation:** Ilona Schubert.

**Formal analysis:** Ilona Schubert, Peter C. Strohm, Jörn Zwingmann.

**Investigation:** Ilona Schubert, Peter C. Strohm, Jörn Zwingmann.

**Methodology:** Jörn Zwingmann.

**Project administration:** Ilona Schubert, Dirk Maier, Jörn Zwingmann.

**Software:** Ilona Schubert.

**Supervision:** Peter C. Strohm, Jörn Zwingmann.

**Validation:** Ilona Schubert, Jörn Zwingmann.

**Visualization:** Ilona Schubert.

**Writing – original draft:** Ilona Schubert.

**Writing – review & editing:** Dirk Maier, Jörn Zwingmann.
